# Changes in Adhesion and the Expression of Adhesion Molecules in PBMCs after Aneurysmal Subarachnoid Hemorrhage: Relation to Cerebral Vasospasm

**DOI:** 10.1007/s12975-023-01136-6

**Published:** 2023-02-23

**Authors:** Gonzalo Revilla-González, Lourdes María Varela, Zaida Ruiz de Azua-López, Rosario Amaya-Villar, María Rosa Pezzotti, María José Castro, Juan Ureña, María del Carmen González-Montelongo, Antonio Castellano

**Affiliations:** 1 Instituto de Biomedicina de Sevilla (IBiS), Hospital Universitario Virgen del Rocío/CSIC/Universidad de Sevilla, Sevilla, Spain; 2 Dpto. Fisiología Médica y Biofísica, Facultad de Medicina, Universidad de Sevilla, Sevilla, Spain; 3 UGC de Cuidados Intensivos, Hospital Universitario Virgen del Rocío, Sevilla, Spain; 4 Unidad de Investigación, Instituto de Investigación e Innovación Biomédica de Cádiz (INiBICA), Hospital Universitario Puerta del Mar, Cádiz, Spain

**Keywords:** Aneurysmal subarachnoid hemorrhage (aSAH), Peripheral blood mononuclear cells (PBMCs), Vasospasm (VSP), Adhesion to the endothelium, Adhesion molecules

## Abstract

Aneurysmal subarachnoid hemorrhage (aSAH) is a neurovascular disease produced by extravasation of blood to the subarachnoid space after rupture of the cerebral vessels. After bleeding, the immune response is activated. The role of peripheral blood mononuclear cells (PBMCs) in this response is a current subject of research. We have analysed the changes in PBMCs of patients with aSAH and their interaction with the endothelium, focusing on their adhesion and the expression of adhesion molecules. Using an in vitro adhesion assay, we observed that the adhesion of PBMCs of patients with aSAH is increased. Flow cytometry analysis shows that monocytes increased significantly in patients, especially in those who developed vasospasm (VSP). In aSAH patients, the expression of CD162, CD49d, CD62L and CD11a in T lymphocytes and of CD62L in monocytes increased. However, the expression of CD162, CD43, and CD11a decreased in monocytes. Furthermore, monocytes from patients who developed arteriographic VSP had lower expression of CD62L. In conclusion, our results confirm that after aSAH, monocyte count and adhesion of PBMCs increase, especially in patients with VSP, and that the expression of several adhesion molecules is altered. These observations can help predict VSP and to improve the treatment of this pathology.

**Supplementary Information:**

The online version contains supplementary material available at 10.1007/s12975-023-01136-6.

## Introduction

Aneurysmal subarachnoid hemorrhage (aSAH) is a neurovascular pathology in which blood is extravasated into the subarachnoid space due to rupture of cerebral arterial vessels. Its worldwide incidence is 6.1 per 100,000 person-year [1], showing a fatality rate of 32.2 to 44.4% depending on the region. It is estimated that 19% of survivors become disabled for daily work [2], as they can develop cognitive, functional, and behavioral affections [3, 4]. Damages after the onset of rupture are divided into early cerebral injury (ECI, up to 72 h after aSAH) and delayed cerebral injury (DCI, from day 3 to day 14 after aSAH) [5]. Cerebral vasospasm (VSP) may develop 4–14 days after hemorrhage [5]. Recent insights into the pathophysiology of aSAH have indicated that during ECI, cytokine production and the immune system are activated [6, 7]. In the same way, inflammation and activation of the immune system have been associated with DCI [8, 9].

Peripheral blood mononuclear cells (PBMCs) are a group of immune cells composed of lymphocytes and monocytes. Monocytes are involved in the innate response, while lymphocytes are related to the adaptive one. When an aneurysm ruptures, blood is released and hemoglobin accumulates in the subarachnoid space where it is removed, among other cell types, by monocytes. These cells are attracted to the bleeding site and remain there, releasing vasoactive substances [10]. Knowledge of the role of lymphocytes and monocytes in tissue damage and its association with VSP is scarce [11], and conflicting results have been reported. T and B lymphocytes have been shown to be present in the arterial wall of aneurysms [12, 13]. A higher activation state of lymphoid cells has been described in aSAH [14], although an immunodepressive state has also been reported in these patients [15]. Several studies have identified a decrease in the number of lymphoid cells in peripheral blood and an increase in monocyte count [14, 16]. Furthermore, expression studies have reported negative regulation of specific T lymphocyte subpopulation transcripts, while those related to monocytes and neutrophils were upregulated [17, 18].

Cell adhesion molecules play a key role in the regulation of many aspects of immune cell function, including adhesion to the endothelium, cell trafficking into tissues, and cell activation during inflammation [19]. It has long been known that blood spillage into the subarachnoid space stimulates the expression of specific cell adhesion molecules in endothelial cells [20], and more recently, it has been shown that the expression of some adhesion molecules is also altered in leukocytes in the acute phase of aSAH [16]. However, those changes in the adhesion molecules in the PBMCs after aSAH have been poorly studied. Previous results from our laboratory demonstrated an increase in RhoA expression and activity in PBMCs from patients with aSAH who developed VSP [21]. The RhoA/ROCK pathway is known to play an important role in leukocyte adhesion to the endothelium and transmigration [22, 23]. Therefore, the purpose of this work was to investigate aSAH-induced alterations in PBMCs through changes in their subpopulations, adhesion to the endothelium, the expression profile of their adhesion molecules, and their relationship with VSP.

## Materials and Methods

### Patients and Healthy Control Subjects

Patients with aSAH admitted to the Intensive Care Unit of the University Hospital Virgen del Rocío (Seville, Spain) were evaluated to participate in this study. Patients with evidence of bleeding, by computed tomography, were enrolled within the first 24 h after the appearance of symptoms. Those who had traumatic SAH, SAH secondary to arteriovenous malformations, blood neoplasias or dyscrasias, previous SAH events, central nervous system pathologies, kidney failure, long-term monitoring difficulties, or with dyslipidemic or antihypertensive treatment were excluded. A group of healthy control subjects without SAH was also included.

This study was carried out with the approval of the Ethics Committee of the Virgen del Rocío University Hospital (C.I. 0586-N-16). The informed consent was signed by the relatives of legal representatives. Due to the fact that the origin of the samples was human, ethical research principles were fulfilled following the Declaration of Helsinki and the Belmont report. This study also adhered to legal provisions that govern human research and the Spanish Organic Law 3/2018 for the protection of personal data and the guarantee of digital rights.

### aSAH Severity and Diagnosis of VSP

Hemodynamic and metabolic resuscitation was performed after admission, and then the Glasgow Coma Scale (GCS), Hunt-Hess, World Federation of Neurological Surgeons (WFNS), and modified Fisher scales were used, within the first 24 h, to assess the condition of the patients. GCS was used to evaluate consciousness 24 h after resuscitation, Hunt-Hess and WFNS scales were used to evaluate clinical severity after aSAH, and the modified Fisher scale evaluated the risk of delayed ischemia. The presence of VSP was evaluated using three different diagnostic criteria: sonographic, clinical, and arteriographic. The occurrence of vasospasm was assessed over a period of 14 days after aneurysmal subarachnoid hemorrhage. A more detailed description of the evaluation and diagnosis of VSP can be found in the “Supplementary information”.

### PBMCs Collection and Cryopreservation

PBMCs were isolated from human blood samples. Samples were obtained from patients within the first 24 h and 5 days (5d) after the appearance of symptoms, and from healthy control subjects at one time. PBMCs were isolated using cell preparation tubes (CPT, BD Vacutainer®) containing a 0.1 M sodium citrate solution. These tubes contain a cell separation medium comprised of a polyester gel and a density gradient liquid (based on ficoll), an accepted technique for mononuclear cell separation. The tubes were inverted 8–10 times and the samples were immediately processed following the manufacturer’s instructions. Subsequently, the PBMCs were frozen in the presence of their own plasma + 10% DMSO using a slow-freezing dispositive at − 80 °C and, eventually, cryopreserved in liquid nitrogen.

### Thawing and Maintenance of Cells in Culture

The frozen vials with 1 mL of PBMCs were warmed at 37 °C until thawed (no longer than 1 min). Subsequently, the vial contents were transferred to Falcon tubes with 8 mL of tempered RPMI-1640 medium (GIBCO) supplemented with 10% FBS, 1% glutamine, and 1% penicillin/streptomycin. Vials were rinsed with 1 mL of the supplemented medium that was also transferred to the Falcon tubes. Subsequently, cells were centrifuged at 400 × *g* for 10 min, discarding the supernatant and resuspending cells in 1 mL of supplemented medium. After this process, PBMCs were used in the experiment at the needed concentration. In our hands, the freezing–thawing process does not affect the viability of the recovered cells or the percentages of the different PBMS populations.

HUVEC (5 × 10^4^ cells) were seeded in 25 cm^2^ culture flasks with 5 mL of EBM medium and EGM supplements (Lonza BulletKit) and incubated at 37 °C, 5% CO_2_ and 90% humidity in a cell incubator. The growth medium was changed no longer than 16 h after thawing to discard DMSO and then changed every 2 days. Cells between passages 2 and 7 were used in the experiments.

### PBMCs-Endothelium Adhesion Assay

The adhesion of PBMCs to the endothelium was analyzed with the CytoSelect™ leukocyte-endothelium adhesion assay (Cell Biolabs Inc.) using 96-well cell culture plates. The required wells were treated with 100 μL of gelatin per well and incubated for 1 h at 37 °C. Subsequently, the wells were washed twice with PBS and HUVEC cells were seeded, in 200 μL of supplemented EBM medium, at 5 × 10^4^ cells per well. Cells were cultured for 48 h until a monolayer was formed. For adhesion assays, HUVECs were treated with 50 ng/mL TNFα for 12 h prior to the assay. Just before the experiment, PBMCs were resuspended at 10^6^ cells/mL in RPMI-1640 medium supplemented with 0.5% BSA and stained with LeukoTracker for 1 h at 37 °C. The cells were then washed twice with RPMI-1640 medium supplemented with 0.5% BSA. The wells with HUVEC were washed with the same medium and PBMCs were added and cocultured for 1 h. Finally, the wells were carefully washed to remove non-adherent cells, and lysis buffer was added. Fluorescence was evaluated on a spectrophotometer.

### Immunocharacterization of PBMCs

Immunocharacterization of the PBMCs (circulating and adhered) was performed by flow cytometry. Adhered PBMCs were dissociated with Cell Dissociation Buffer Enzyme-Free PBS-based (Gibco) and centrifuged at 400 × *g* for 10 min. The PBMCs were stained for 30 min at room temperature with the following panel of mouse anti-human antibodies (BD Bioscience) at the recommended concentration: anti-CD45-APC-H7, anti-CD11b-APC, anti-CD3-PE-Cy7, anti-CD19-BV605, anti-CD11a-APC-R700, anti-CD162-BV421, anti-CD49d-PE-CF594, anti-CD62L-BV711, (1:100) anti-CD43-PE and 7AAD. Cells were washed, resuspended in 400 μL of PBS, and directly used for flow cytometry. Data were collected on a BD FACS LRS II Fortessa equipped with four lasers. The adhesion molecule expression was quantified by measuring the median fluorescent intensity (MFI) of each label-conjugated antibody and then calculating the mean MFI for each marker. BD FACS Diva software v. 8.0 was used for data acquisition and FlowJo v. 10.6.1 was used for data analysis. The following gating strategy was used (see Supplementary Fig. 1): first, identification of the cells of interest was performed by SSC-A and FSC-A, and the selected events were filtered by FSC-A and FSC-H to obtain singlets. Subsequently, viability was evaluated by exclusion of 7-AAD. Viable PBMCs were evaluated by SSC-A and expression of CD45. Lymphocytes and monocytes were divided by SSC-A and exclusion of CD11b. Finally, lymphocyte subpopulations were identified by expression of CD3 and CD19.

### Statistical Analysis

Data are expressed as mean ± SEM except for characterization of the PMBC subsets and severity scales where the median and interquartile range (P_25_-P_75_) were used. Normality was determined with Shapiro–Wilk and homoscedasticity was evaluated with the Levene test. Differences were evaluated by ANOVA or Kruskal–Wallis test followed by a Student’s *t* test or Mann–Whitney *U* test when corresponding. Comparisons between experiments from the same patients were assessed by paired *t* test. Furthermore, correlation analyses were performed using the Pearson or Spearman test when corresponding. To perform the analysis, the Statistical Package for the Social Sciences (SPSS) v26 (IBM) was used. Graphics were prepared with SigmaPlot v14 (Jandel Scientific) and Canvas 12 (ACD System of America Inc.).

## Results

### Demographic and Clinical Characteristics of Healthy Control Subjects and Patients with aSAH

Demographic and clinical data were collected from patients with aSAH and healthy control subjects (Table 1).

**Table 1:** Demographic data of healthy control subjects and patients with aSAH, and classification of patients using different evaluation scales

Variable		Control (*n* = 25)	aSAH (*n* = 28)
Age, mean (SEM)		42.04 (2.13)	56.75 (2.08)
Sex, n (%)	Men	14 (56)	9 (32.1)
	Women	11 (44)	19 (67.9)
Smoker, n (%)	No	21 (91.3)	12 (44.4)
	Yes	2 (8.7)	15 (55.6)
Alcohol intake, n (%)	No	16 (69.6)	25 (89.3)
	Yes	7 (30.4)	3 (10.7)
Hypertension, n (%)	No	21 (91.3)	12 (42.9)
	Yes	2 (8.7)	16 (57.1)
Grading of aSAH severity, Median (IQR, P25-P75)			
GCS	13 [10.5–15]		
Hunt-Hess	3 [2–4]		
WFNS	2.5 [2–4]		
Modified Fisher	4 [4–4]		
VSP evaluation, Frequency (%)			
Sonographic	16/28 (57.1%)		
Clinical	10/28 (35.7%)		
Arteriographic	13/28 (46.4%)		

A clinical evaluation was performed to study the condition and prognosis of the patients at the time of admission. The classification of the patients according to the different evaluation scales is summarized in Table 1. Most of the patients had a high degree of consciousness at admission according to GCS. Furthermore, a high percentage of patients had a medium grade on the Hunt-Hess and WFNS scales. However, a large group of patients was at high risk of developing cerebral ischemia after aSAH, according to the modified Fisher scale.

### Immunological Characterization of Circulating PBMCs and its Relationship to VSP

**Fig. 1:**
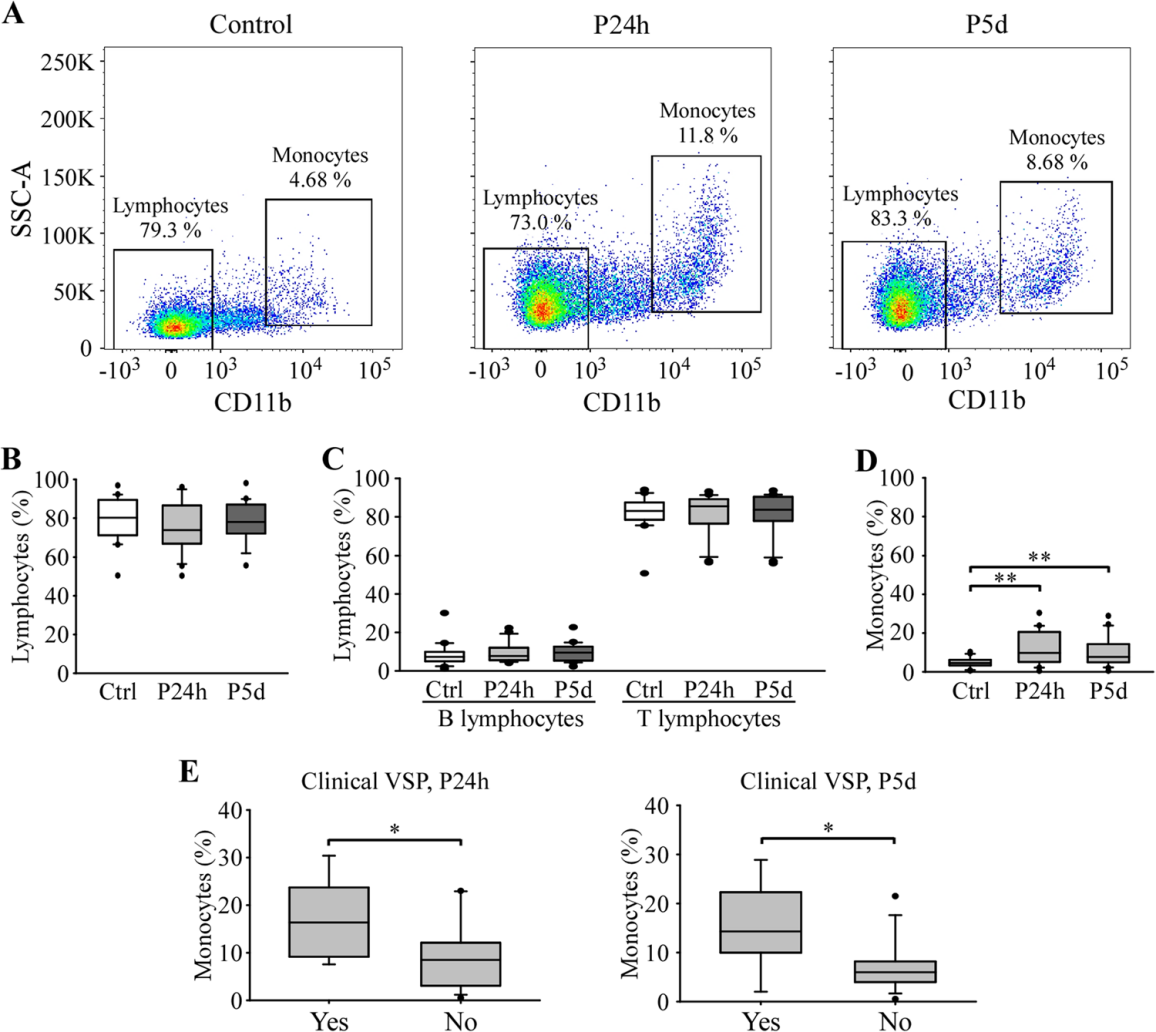
Statistical analysis of the percentages of lymphocytes and monocytes in circulating PBMCs in control subjects and patients. **A** Representative gates showing the selection and quantification of lymphocyte and monocyte populations by SSC-A and the exclusion of CD11b. Quantification of the percentages of lymphocytes (**B**), B and T lymphocytes (**C**) and monocytes (**D**). **E** Statistical analysis of the relationship between the percentage of monocytes and the presence of clinical VSP. *n* = 20 control subjects and 21 patients. *, ** *P* ≤ 0.05, 0.01

Differences in circulating PBMC subpopulations between patients and control subjects were analyzed by flow cytometry. The results of the immunological characterization are summarized in Fig. 1. The representative gating strategy for each group is represented in Fig. 1A. No significant differences were observed in the percentages of total lymphocytes, nor in the subpopulations of lymphocytes between control subjects and patients (Fig. 1B and C). However, the percentage of monocytes increased significantly in patients 24 h and 5 days after aSAH, compared to control subjects (Fig. 1D). Subsequently, we analyzed whether there were differences in the percentages of monocytes between patients with VSP and those without VSP, using three different diagnostic criteria for the presence of VSP: sonographic, clinical and arteriographic (Table 1). Figure 1E shows that patients who developed clinical VSP presented a significant increase in the number of circulating monocytes 24 h and 5 days after bleeding compared to patients without VSP. No significant differences were found when using sonographic or arteriographic criteria.

### Adhesion of PBMCs to Endothelial Cells

**Fig. 2:**
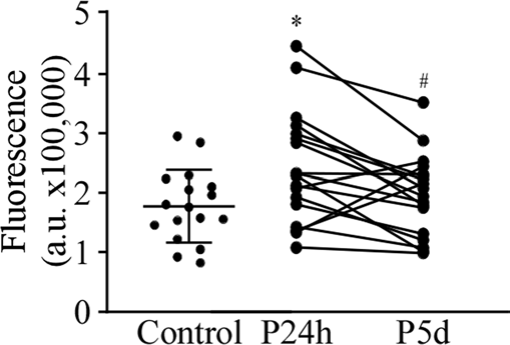
Adhesion of PBMCs to endothelial cells. Statistical analysis of the adhesion of PBMCs to HUVECs. The HUVECs were stimulated with 50 ng/mL TNFα for 12 h before the assay, and the PMBCs were stained with a fluorescent marker. Fluorescence was measured in arbitrary units (a.u.). *n* = 17 control subjects and 18 patients. * *P* ≤ 0.05 vs. control; ^#^*P* ≤ 0.05 vs. P24h

The adhesion of leukocytes to the vascular endothelium is a hallmark of the inflammatory process; however, this process has not been analyzed in detail in the aSAH. Therefore, we have studied the adhesion of PBMCs to the endothelium using, as a substrate, a monolayer of HUVEC preincubated with 50 ng/mL TNFα for 12 h. The PBMCs of the patients, in the first 24 h after aSAH, showed a significant increase in the adhesion to HUVEC compared to control subjects (Fig. 2). However, on day 5 after bleeding, this increase was not observed.

### Immunocharacterization of Adhered PBMCs and its Relation to VSP

**Fig. 3:**
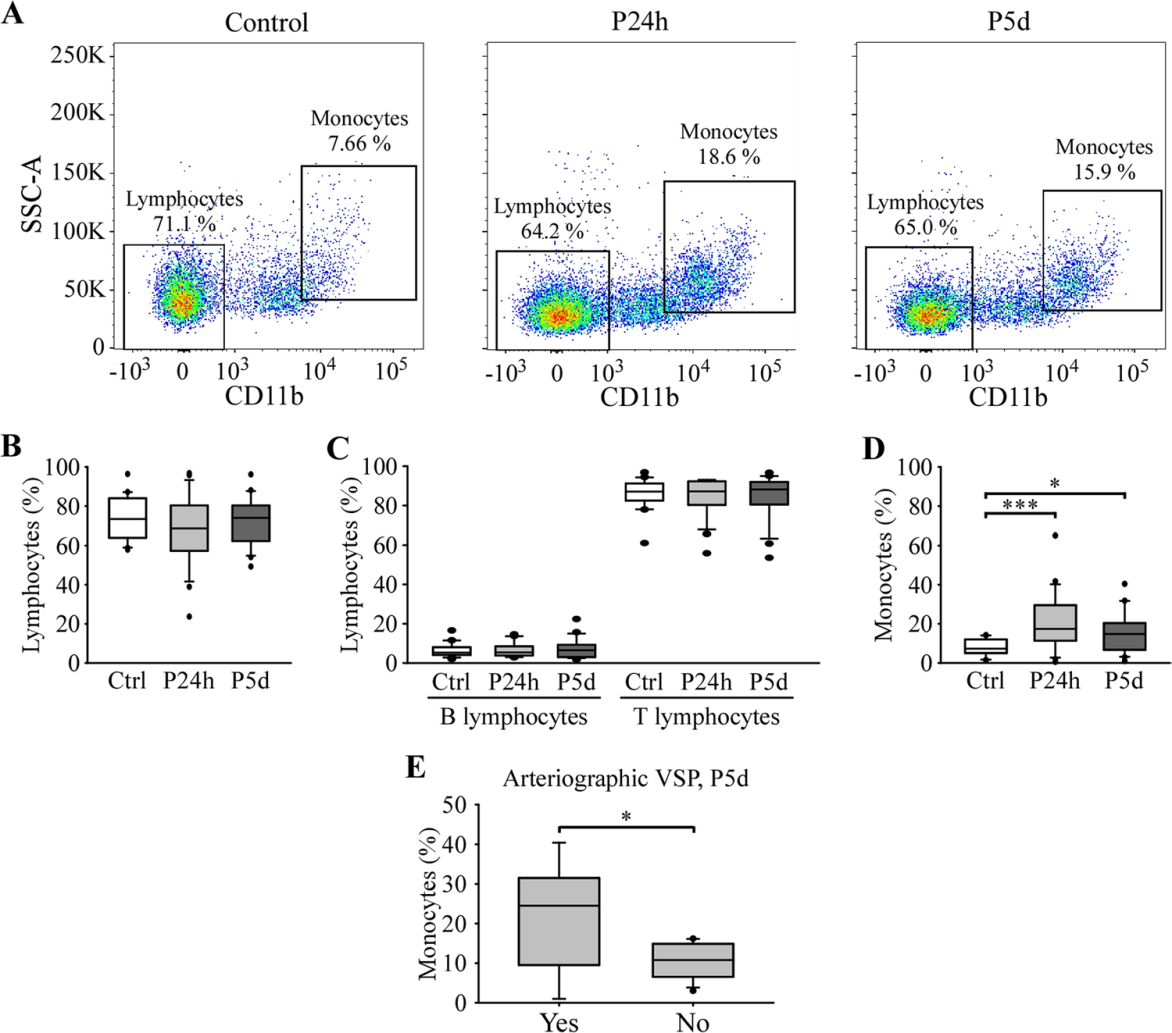
Statistical analysis of the percentages of lymphocytes and monocytes in adhered PBMCs of control subjects and patients. **A** Representative gates used for quantification of lymphocyte and monocyte populations by SSC-A and exclusion of CD11b. Quantification of the percentages of lymphocytes (**B**), B and T lymphocytes (**C**), and monocytes (**D**). **E** Relationship between the percentage of monocytes and the presence of arteriographic VSP. *n* = 20 control subjects and 21 patients. *, *** *P* ≤ 0.05, 0.001

To identify the subtypes of PBMCs that interact with HUVECs, we addressed their immunocharacterization. Therefore, we carried out the adhesion assay, applied a dissociation protocol (see the “Materials and methods” section) to recover the PBMCs that had adhered to the endothelial cells, and, subsequently, performed flow cytometry (Fig. 3). The representative gates for each group are represented in Fig. 3A. As in the case of circulating PBMCs (Fig. 1), no significant differences were observed in the percentage of total lymphocytes or their subpopulations between control subjects and patients (Fig. 3B and C), while the adhered monocyte population increased significantly in patients 24 h and 5 days after aSAH compared to control subjects (Fig. 3D). As with circulating PBMCs, we analyzed whether there were differences in the percentage of monocytes between patients with VSP and those without VSP. Interestingly, patients who developed arteriographic VSP had a significantly increased number of adhered monocytes 5 days after aSAH, compared to patients without VSP (Fig. 3E). This observation is similar to what has been reported in the case of circulating monocytes (Fig. 1E).

### Comparison of the Expression Profile of Adhesion Molecules Between Circulating and Adhered PBMCs

**Fig. 4:**
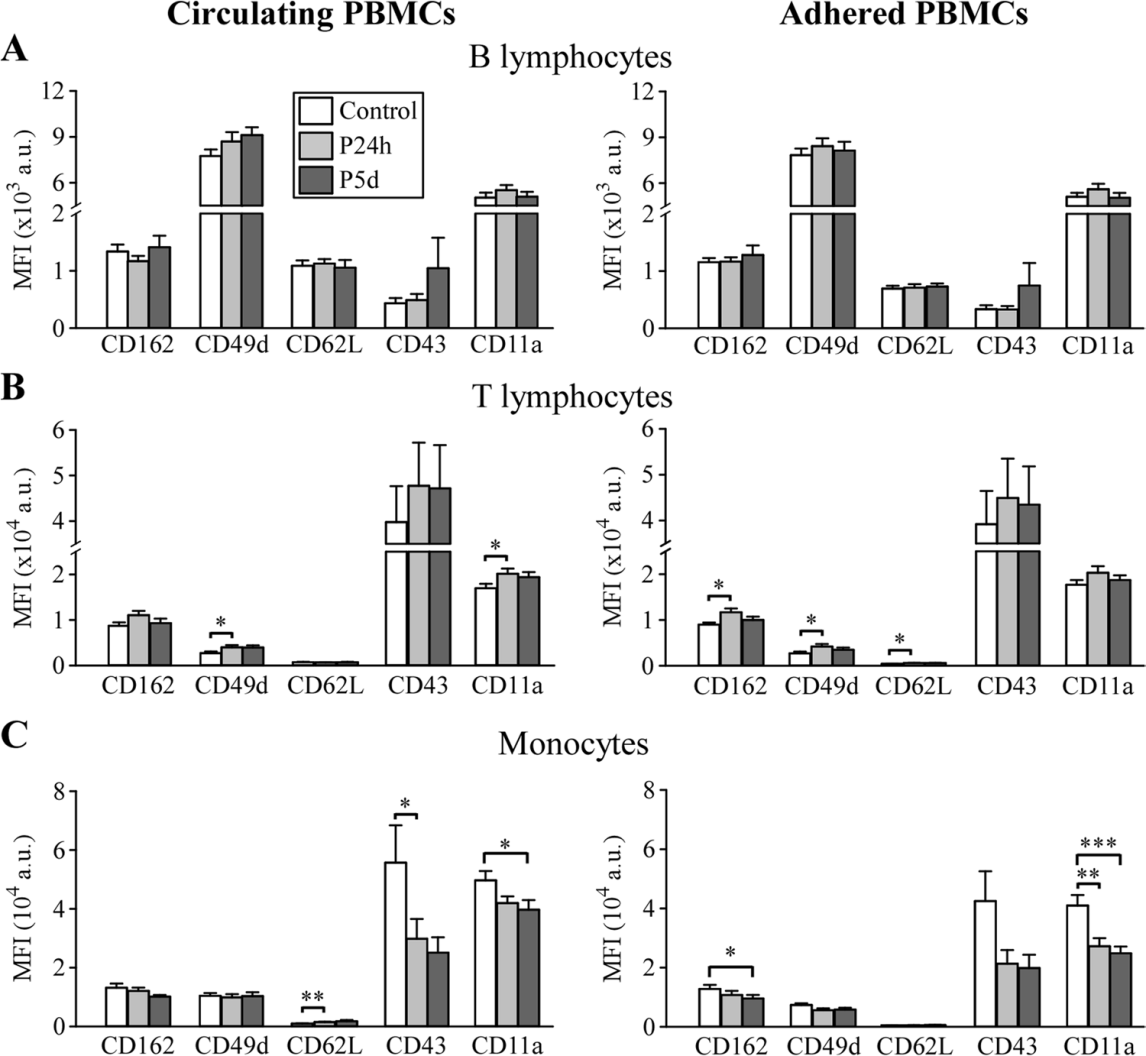
Expression of surface adhesion molecules in circulating and adhered lymphocyte and monocyte populations. Quantification of adhesion molecules by MFI using flow cytometry. **A** B lymphocytes, **B** T lymphocytes and **C** monocytes. *n* = 20 control subjects and 21 patients. *, **, *** *P* ≤ 0.05, 0.01, 0.001

The interaction between leukocytes and the endothelium is mediated by adhesion molecules located on the cell surfaces. Although activation of the inflammatory response after aSAH has been described, little is known about the changes in the expression of adhesion molecules in this pathology. For that reason, we have analyzed, in PBMCs, the expression of some adhesion molecules known to be involved in inflammatory processes (Fig. 4). We performed this analysis in both circulating and adhered PBMCs. In general, the expression profiles of the adhesion molecules were similar in both PBMCs. However, some specific changes were observed.

In circulating PBMC, CD49d and CD11a expression was significantly increased in T cells from patients 24 h after aSAH (Fig. 4B, left panel) compared to controls. The increase in CD49d was close to significance 5 days after bleeding (Supplementary Table 1). On the other hand, in circulating monocytes, a significant decrease in CD11a was observed on day 5 and in CD43 expression at 24 h (Fig. 4C, left panel). The decrease in CD11a was close to statistical significance in patients 24 h after bleeding (Supplementary Table 1). Furthermore, a significant increase in CD62L expression was observed in monocytes 24 h after aSAH (Fig. 4C, left panel). Those changes in CD62L and CD43 expression were close to significance on day 5 after bleeding (Supplementary Table 1). No significant changes in the expression of adhesion molecules were observed in B lymphocytes from patients with aSAH.

On the other hand, adhered T cells from patients 24 h after aSAH had a significantly higher expression of CD162, CD49d and CD62L than control T lymphocytes (Fig. 4B, right panel). In adhered monocytes, significant decreases in CD162 expression were observed in patients 5 days after aSAH and in CD11a expression 24 h and 5 days after aSAH (Fig. 4C, right panel). Furthermore, in adhered monocytes, a decrease in the expression of CD49d and CD43 was observed, close to statistical significance, in patients 24 h and 5 days after aSAH. The complete statistical analysis is shown in Supplementary Table 1. Supplementary Fig. 2 shows the statistically significant changes, patient by patient, in the expression of adhesion molecules.

**Fig. 5:**
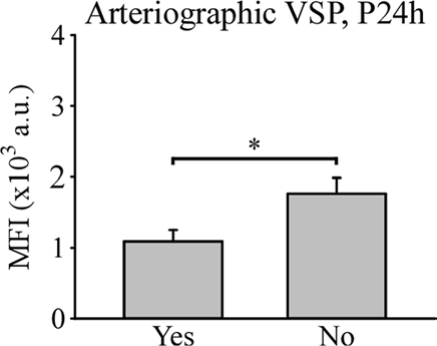
Relationship between CD62L expression in circulating aSAH monocytes, within the first 24 h after bleeding, and the presence of arteriographic VSP. *n* = 21 patients (9 with VSP and 12 without VSP). * *P* ≤ 0.05

To find out whether the expression of adhesion molecules is related to the patient’s condition, we analyzed the correlations between the expression of adhesion molecules and the presence of VSP. CD62L expression, in the first 24 h after aSAH, was significantly lower in circulating monocytes of patients who were eventually diagnosed with arteriographic VSP (Fig. 5).

## Discussion

We have evaluated whether the interaction of PBMCs with the endothelium is altered in patients with aSAH. We demonstrate that PBMCs from patients with aSAH have a higher adhesion to HUVEC, a higher percentage of monocytes, and altered expression of adhesion molecules in their membranes. We also show that patients who developed VSP have a higher number of monocytes than those who did not, and that CD62L expression is lower in monocytes from patients with VSP.

Previous reports have shown a decrease in lymphocyte count in the first 24 h after aSAH [14–16, 24]. Although we have not observed significant differences in the number of circulating lymphocytes or in their subpopulations, between control subjects and patients, our data show a similar tendency (Fig. 1B). On the other hand, we have observed a significant increase in the number of circulating monocytes in patients within the first 24 h, a result similar to that previously described in patients 48 h after aSAH [16]. The monocyte count remained significantly higher on day 5 after aSAH, which is consistent with previous work describing the overexpression of monocyte-related genes in whole blood samples [17, 18]. A similar increase in monocyte count has also been observed in CSF longer after bleeding [25]. We have also observed a higher percentage of circulating and adhered monocytes in patients with clinical and arteriographic VSP, respectively, compared to patients without it. This increase is consistent with recent reports showing an increase in the absolute number of monocytes on day 0 in patients who later suffered from VSP [24, 26] and supports the idea that the increase in the number of monocytes could help early assess the risk of developing VSP in patients with aSAH.

Adhesion of PBMCs to the endothelium, in an in vitro assay, is higher in patients than in control subjects in the first 24 h, although on day 5 that difference in adhesion is no longer observed. This increase in adhesion is primarily due to the increase in the number of monocytes in patients with aSAH. However, in our adhesion assays, a higher percentage (although not statistically significant) of monocytes from patients than from controls adhered to endothelial cells, suggesting that monocytes are the group of cells that primarily increase adhesion in patients. On the other hand, changes in adhesion may reflect alterations in the expression of adhesion molecules in PBMCs after hemorrhage. Thus, we have analyzed changes in the expression of adhesion molecules that are known to be involved in the leukocyte-endothelium interaction [16, 19, 27]. Our experiments show increases in CD49d and CD11a expression in circulating T lymphocytes, an increase in CD62L, and decreases in CD43 and CD11a in circulating monocytes from patients with aSAH. In addition, we have compared the differences in the expression profile of these molecules between circulating PBMCs and the PBMCs that adhered to the endothelium in the in vitro adhesion assay, which to the best of our knowledge has not been addressed before. Although the expression pattern of adhesion molecules is similar in circulating and adhered PBMCs, there are some differences in the statistical significance of the changes in adhesion molecules expression. These differences are (i) in adhered lymphocytes, increases in the expression of CD162 and CD62L reach significance, which is not observed in circulating T cells; (ii) the increase in CD11a observed in circulating is not present in adhered T cells; (iii) in adhered monocytes, no changes are observed in the expression of CD62L and CD43; and (iv) the decrease in CD162 reaches significance in patients with aSAH on day 5, a change that is not observed in circulating monocytes. These differences between circulating and adhered PBMCs can help characterize subpopulations of PBMCs with greater adhesion and transmigration capacity.

CD11a (α subunit of LFA-1) is expressed in all types of leukocytes and is important for interactions between these cells and the endothelium. CD11a has been associated with VSP, since treatment with monoclonal antibodies against CD18 and LFA-1 ameliorates blood-induced VSP in animal models of SAH [28, 29] and femoral artery hemorrhage [30], respectively. We have observed an increase in CD11a in T lymphocytes from patients with aSAH, and a similar increase is observed in some inflammatory pathologies, such as rheumatoid arthritis [31, 32]. On the other hand, we have observed a decrease in CD11a in monocytes from aSAH patients, which is consistent with previous reports showing a small, although not significant, decrease in LFA-1 expression in monocytes from these patients [16]. A similar tendency, although not statistically significant, has previously been observed in an experimental mouse model of intracerebral hemorrhage [33]. A decrease in CD11a expression has been reported in alveolar macrophages in “healthy” smokers compared to non-smokers [34], which could suggest that the decrease in CD11a observed in monocytes from patients with aSAH could be due to the effect of smoking. However, we have not observed differences in CD11a expression between smokers and non-smokers in patients with aSAH and, furthermore, there is a significant decrease in CD11a expression in monocytes from non-smoker patients compared to controls (non-smokers). These results suggest that smoking is not responsible for the decrease in CD11a expression observed in patients with aSAH and support the hypothesis that the injury produced by aSAH is the reason for this decrease. On the other hand, a reduction in CD11a expression is observed in monocyte-to-macrophage differentiation [35], therefore, it could suggest that, after bleeding, peripheral blood monocytes are already preparing for differentiation.

CD49d (α4 integrin) plays a critical role in leukocyte trafficking and activation. CD49d expression increased significantly in T lymphocytes from patients 24 h after aSAH, an observation not previously reported in humans. A similar increase has also been described in infiltrated lymphocytes in an animal model of intracerebral hemorrhage [33]. Other studies have reported that the administration of an anti-CD49d antibody reduces the number of infiltrated lymphocytes in animal models of cerebral infarction [33, 36–38]. Thus, CD49d may represent a potential therapeutic target for the treatment of aSAH.

CD43 (also known as leukosialin and sialophorin) is a surface sialoglycoprotein expressed in leukocytes that is involved in the adhesion and migration of PBMCs to inflamed tissue [27, 39] and activation mechanisms [40]. We have observed a significant decrease in CD43 expression in monocytes from patients 24 h after aSAH. This observation is consistent with a previous report in which CD43 expression decreased after exposure of THP-1 cells (a monocyte cell line) to TNFα and INFγ [41]. Furthermore, in a mouse model, stroke has been shown to mobilize immature pro-inflammatory Ly6C^hi^CD43^lo^ monocytes that infiltrate ischemic tissue on their way to the core of the lesion [42]. On the other hand, it is known that a decrease in CD43 expression is correlated with an activated phenotype in neutrophils [40]. Considering that it is a different cell type, it is plausible to think that the decrease in CD43 expression that we observed in aSAH monocytes may also indicate their activation.

CD162 (PSGL-1) is a sialoglycoprotein involved in adhesion and inflammatory processes that is expressed in lymphocytes and monocytes [43, 44]. Its expression has a different behavior depending on the PBMC subset. CD162 increases in adhered lymphocytes in patients with aSAH, suggesting a higher transmigration capacity. However, we have observed a decrease in CD162 in adhered monocytes. Other authors have reported a similar result [16]. Downregulation of CD162 in monocytes has also been observed in other inflammatory diseases, such as systemic inflammation [45] and rheumatoid arthritis [46]. However, PSGL-1 expression increases in monocytes after ischemic stroke [47]. These observations suggest that CD162 expression in PBMCs may change depending on the pathophysiological scenario.

CD62L (L-selectin) is involved in T lymphocyte homing processes in lymph nodes or inflamed tissues and in monocyte adhesion [48, 49]. Our experiments show significant increases in CD62L (L-selectin) expression in adhered T cells and circulating monocytes from patients 24 h after aSAH. An increase in L-selectin expression in monocytes has also been reported in an animal model of intracerebral hemorrhage [33]. This increase in CD62L expression could be due to the increase in pro-inflammatory monocytes induced by the inflammatory process [50]. Finally, we have observed that patients who developed cerebral VSP had significantly lower CD62L expression than patients without VSP. Our results are consistent with previous results showing a reduction in CD62L detection by flow cytometry due to cleavage (shedding) of the extracellular domain of L-selectin in activated monocytes [50, 51]. These observations could represent a promising marker for VSP. However, more studies, including a larger number of patients, will be necessary to prove this idea.

In summary, our observations indicate that, after bleeding, there is an increase in the number of monocytes in peripheral blood in patients with aSAH, and that this increase is correlated with the subsequent appearance of VSP. Furthermore, PBMCs increase their adhesion to the endothelium and the expression of adhesion molecules on their membranes is altered. Additionally, CD62L expression is inversely correlated with the presence of VSP in patients with aSAH. Therefore, the study of serial changes in the expression of adhesion molecules on the PBMC membrane can help to find a VSP biomarker after aSAH and could help improve the diagnosis and treatment of this pathology.

### Supplementary Information

Below is the link to the electronic supplementary material.

Supplementary file1 (DOCX 9.29 MB)

## Data Availability

The data that support the findings of this study are available from the corresponding authors upon reasonable request.
