# Supplementary material for: Changes in Adhesion and the Expression of Adhesion Molecules in PBMCs after Aneurysmal Subarachnoid Hemorrhage: Relation to Cerebral Vasospasm
Source: Transl Stroke Res. 2023 Feb 23;15(2):378–87. doi: 10.1007/s12975-023-01136-6 (PMC10891186; doi:10.1007/s12975-023-01136-6)

**Supplementary material**

**Changes in adhesion and the expression of adhesion molecules in PBMCs after Aneurysmal Subarachnoid Hemorrhage: relation to cerebral vasospasm**

Gonzalo Revilla-González^1,2^, Lourdes María Varela^1,2^, Zaida Ruiz de Azua-López^1,3^, Rosario Amaya Villar^1,3^, María Rosa Pezzotti^1,2^, María José Castro^1^, Juan Ureña^1,2^, María del Carmen González-Montelongo^1,2,4^* and Antonio Castellano^1,2,^*

^1^Instituto de Biomedicina de Sevilla (IBiS), Hospital Universitario Virgen del Rocío/CSIC/ Universidad de Sevilla, Sevilla, Spain.

^2^Dpto. Fisiología Médica y Biofísica, Facultad de Medicina, Universidad de Sevilla, Spain.

^3^UGC de Cuidados Intensivos, Hospital Universitario Virgen del Rocío, Sevilla, Spain.

^4^Unidad de Investigación, Instituto de Investigación e Innovación Biomédica de Cádiz (INiBICA), Hospital Universitario Puerta del Mar, Cádiz, Spain.

*Correspondence to: acastell@us.es, Tel.: +(34)-955-923059 (A.C.); mcarmen.gonzalez@inibica.es, Tel.: (34)-490007 / 956245007 (M.C.G-M.)

**Materials and methods**

**Diagnosis of VSP**

Vasospasm diagnosis was performed via clinical symptoms, transcranial Doppler, or angiography between days 0 to 14 after the bleeding event. Symptomatic vasospasm was defined as worsening of the level of consciousness of ≥2 points on the Glasgow Coma Scale, or by the appearance of a focal defect (such as hemiparesis, aphasia, apraxia, hemianopia, or neglect) between days 4 and 14 after the bleeding event, not attributable to rebleeding, hematoma, hydrocephalia, metabolic alterations, infection, or oversedation. Transcranial Doppler exploration (Doppler Multidop-P, DWL) was performed with a 2-MHz ultrasound probe through the temporal window, in both hemispheres. The first study was carried out on the third day, and every day between days 3 and 14 after aSAH, and beyond this date if the transcranial Doppler recording was not normal. Transcranial Doppler vasospasm or sonographic vasospasm was defined as the mean flow velocity (MV) in any vessel of anterior cerebral circulation ≥120 cm/s. If the MV obtained reached 120 cm/s, the Lindegaard index was calculated by simultaneously recording the homolateral extracranial internal carotid artery at the submandibular site and the MV of the middle cerebral artery. The MV middle cerebral artery/MV extracranial internal carotid artery ratio of >3 was classified as sonographic vasospasm. Patients with MV >120 cm/s and Lindegaard index <3 were diagnosed with increased cerebral blood flow. Using digital subtraction angiography, angiographic vasospasm was defined as a reduction in the diameter of the cerebral artery by more than two-thirds of its baseline caliber.

**Supplemental Table 1.** Statistical analysis of the expression of surface adhesion molecules in circulating and adherent lymphocytes and monocytes. n= 20 control subjects and 21 patients. *, **, *** *P*≤ 0.05, 0.01, 0.001.

|  |  | **CD162** | **CD49d** | **CD62L** | **CD43** | **CD11a** |
| --- | --- | --- | --- | --- | --- | --- |
| **Circulating B lymphocytes** | **Ctrl vs. P24h** | 0.484 | 0.230 | 0.411 | 0.735 | 0.297 |
|  | **Ctrl vs. P5d** | 0.835 | 0.087 | 0.175 | 0.575 | 0.958 |
|  | **P24h vs. P5d** | 0.571 | 0.308 | 0.213 | 0.706 | 0.538 |
| **Circulating T lymphocytes** | **Ctrl vs. P24h** | 0.095 | **0.046 *** | 0.927 | 0.735 | **0.042 *** |
|  | **Ctrl vs. P5d** | 0.938 | 0.052 | 0.335 | 0.907 | 0.110 |
|  | **P24h vs. P5d** | 0.134 | 0.943 | 0.320 | 0.642 | 0.642 |
| **Circulating monocytes** | **Ctrl vs. P24h** | 0.696 | 0.686 | **0.006 **** | **0.050 *** | 0.054 |
|  | **Ctrl vs. P5d** | 0.197 | 0.789 | 0.064 | 0.068 | **0.035 *** |
|  | **P24h vs. P5d** | 0.346 | 0.890 | 0.840 | 0.920 | 0.572 |
| **Adherent B lymphocytes** | **Ctrl vs. P24h** | 0.784 | 0.368 | 0.834 | 0.938 | 0.557 |
|  | **Ctrl vs. P5d** | 0.958 | 0.865 | 0.629 | 0.876 | 0.804 |
|  | **P24h vs. P5d** | 0.950 | 0.624 | 0.814 | 0.960 | 0.232 |
| **Adherent T lymphocytes** | **Ctrl vs. P24h** | **0.014 *** | **0.035 *** | **0.047 *** | 0.303 | 0.150 |
|  | **Ctrl vs. P5d** | 0.335 | 0.220 | 0.127 | 0.855 | 0.486 |
|  | **P24h vs. P5d** | 0.102 | 0.343 | 0.734 | 0.450 | 0.376 |
| **Adherent monocytes** | **Ctrl vs. P24h** | 0.106 | 0.053 | 0.958 | 0.085 | **0.004 **** |
|  | **Ctrl vs. P5d** | **0.025 *** | 0.082 | 0.309 | 0.078 | **0.000 ***** |
|  | **P24h vs. P5d** | 0.443 | 0.816 | 0.333 | 0.734 | 0.511 |

**Supplemental Figure 1.** Gating strategy for the immunocharacterization of PBMCs. (A) Identification of the cells of interest performed by SSC-A and FSC-A. (B) Singlets filter by FSC-A and FSC-H. (C) Alive cells by exclusion of 7-AAD. (D) Identification of PBMCs by SSC-A and expression of CD45. (E) Identification of lymphocytes and monocytes by SSC-A and expression of CD11b. (F) Identification of lymphocyte subpopulations by expression of CD3 and CD19.

**
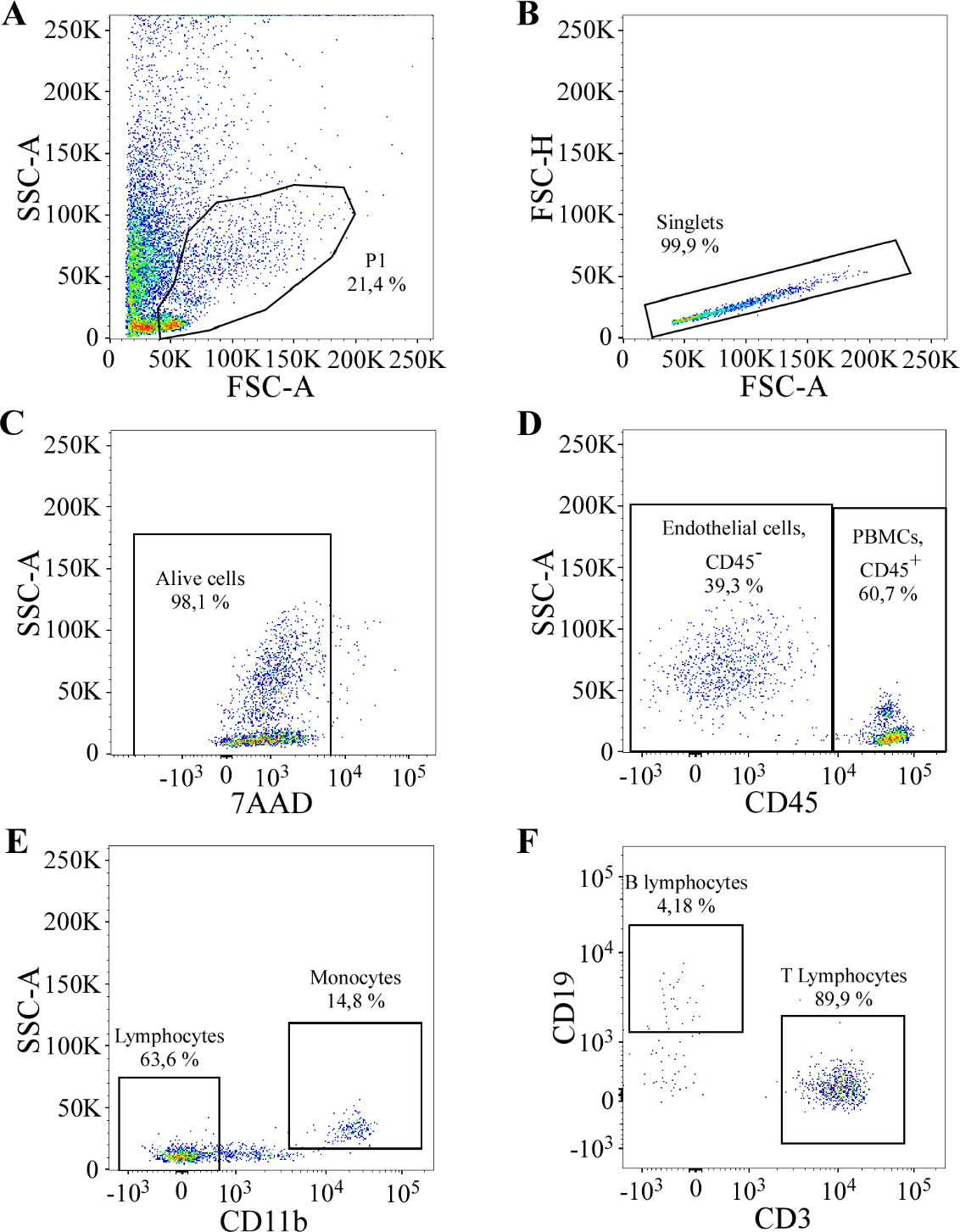
**

**Supplemental Figure 2.** Expression of surface adhesion molecules in circulating and adhered T lymphocytes and monocytes (these data are the same that are shown on Figure 4 of the manuscript, but shown patient by patient). Only statistically significant changes in the adhesion molecules are presented. Comparisons between experiments from the same patients were assessed by paired t test (* *P*≤ 0.05 *vs*. control; ^#^*P*≤ 0.05 *vs*. P24h).


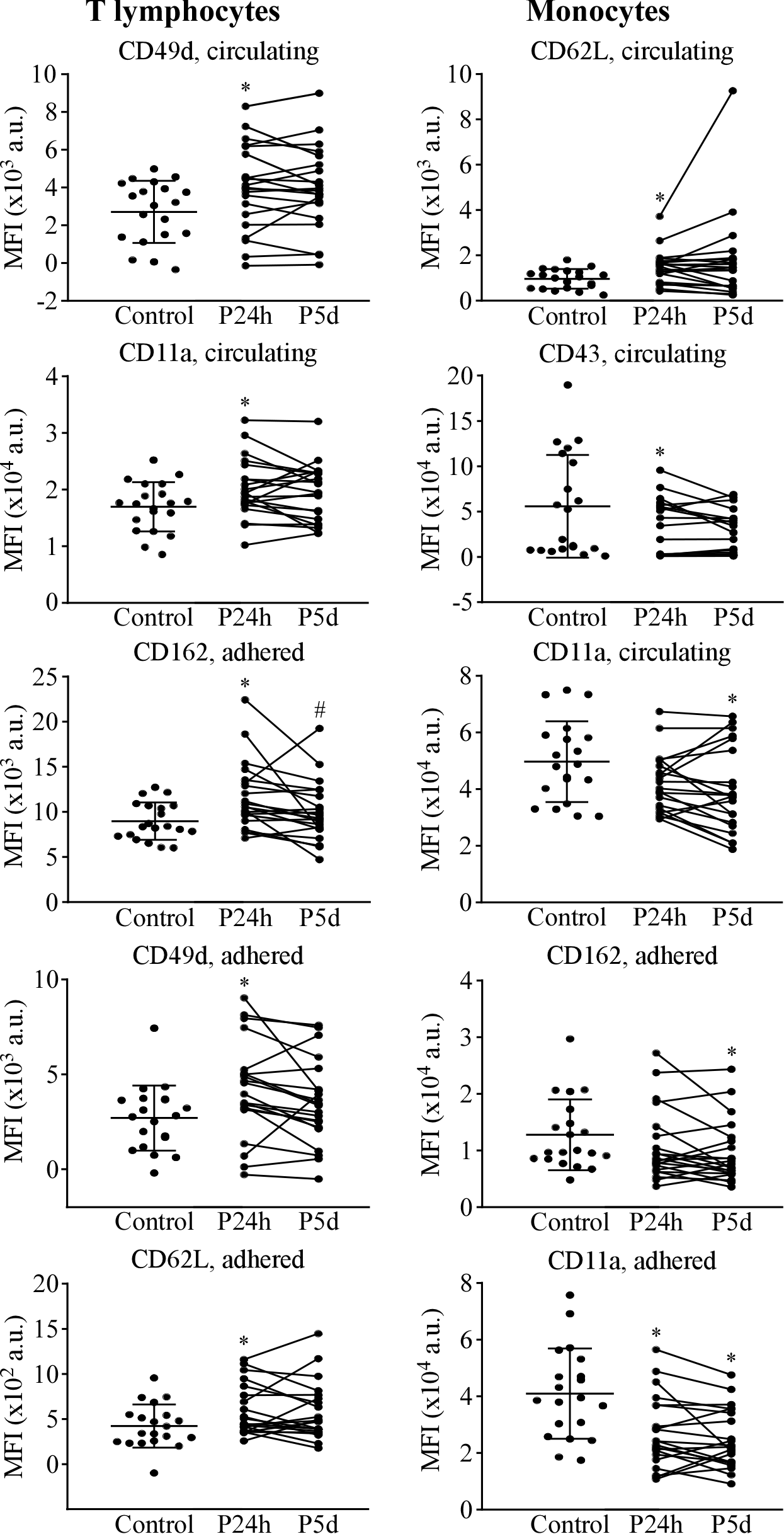

Supplement: Supplementary file 1 — Supplementary file1 (DOCX 9.29 MB) [file 12975_2023_1136_MOESM1_ESM.docx]
